# Chikungunya virus transmission between *Aedes albopictus* and laboratory mice

**DOI:** 10.1186/s13071-016-1838-1

**Published:** 2016-10-19

**Authors:** Leon E. Hugo, Natalie A. Prow, Bing Tang, Greg Devine, Andreas Suhrbier

**Affiliations:** 1 Inflammation Biology, QIMR Berghofer Medical Research Institute, Brisbane, Queensland 4029 Australia; 2 Mosquito Control, QIMR Berghofer Medical Research Institute, Brisbane, Queensland 4029 Australia

**Keywords:** Chikungunya virus, *Aedes albopictus*, Mouse model

## Abstract

**Background:**

Chikungunya virus (CHIKV) is a mosquito-borne alphavirus associated with epidemics of acute and chronic arthritic disease in humans. *Aedes albopictus* has emerged as an important new natural vector for CHIKV transmission; however, mouse models for studying transmission have not been developed.

**Methods:**

*Aedes albopictus* mosquitoes were infected with CHIKV via membrane feeding and by using infected adult wild-type C57BL/6 mice. Paraffin sections of infected mosquitoes were analysed by immunofluorescent antibody staining using an anti-CHIKV antibody. CHIKV-infected mosquitoes were used to infect adult C57BL/6 and interferon response factor 3 and 7 deficient (IRF3/7^-/-^) mice.

**Results:**

Feeding mosquitoes on blood meals with CHIKV titres > 5 log_10_CCID_50_/ml, either by membrane feeding or feeding on infected mice, resulted in  ≥ 50 % of mosquitoes becoming infected. However, CHIKV titres in blood meals  ≥ 7 log_10_CCID_50_/ml were required before salivary glands showed significant levels of immunofluorescent staining with an anti-CHIKV antibody. Mosquitoes fed on blood meals of 7.5 (but not 5.9) log_10_CCID_50_/ml were able efficiently to transmit virus to adult C57BL/6 and IRF3/7^-/-^ mice, with the latter mice showing overt signs of arthritis post-infection.

**Conclusions:**

The results provide a simple in vivo model for studying transmission of CHIKV from mosquitoes to mammals and also argue against a resistance barrier to CHIKV infection in adult mice.

**Electronic supplementary material:**

The online version of this article (doi:10.1186/s13071-016-1838-1) contains supplementary material, which is available to authorized users.

## Background

Chikungunya virus (CHIKV) belongs to a group of mosquito-borne arthritogenic alphaviruses that include the primarily Australian Ross River and Barmah Forest viruses, the African o’nyong-nyong virus, the Sindbis group of viruses and the South American Mayaro virus [1]. The largest documented outbreak of CHIKV disease ever recorded began in 2004 in Africa and spread across the Indian Ocean to Asia, east to Papua New Guinea and several pacific islands, with small outbreaks also seen in Europe. In late 2013 the epidemic reached the Americas, spreading through the Caribbean, Central and South America, with autochthonous transmission also reported in the USA [2, 3]. Millions of cases have been reported.

The traditional vector for CHIKV has been *Aedes aegypti,* and this mosquito species was and remains the main vector in East Africa, the Caribbean and South America. However, the recent epidemic was also associated with efficient CHIKV transmission by *Aedes albopictus* (the so-called Asian tiger mosquito), particularly in the Indian Ocean, West Africa, Europe and Papua New Guinea, with transmission in Asia involving both species. The East/Central/South African (ECSA) genotype of CHIKV developed a mutation in the E1 envelope gene (Alanine 226 to Valine V), which permitted efficient transmission by *Aedes albopictus* [4, 5], a highly anthropophilic and geographically widespread mosquito species [6].

Herein we explore the requirements for transmission of CHIKV (using a Reunion Island isolate with the A226V mutation) between *Aedes albopictus* and mice, and provide the parameters required to establish efficient mosquito-mediated transmission to adult wild-type and interferon response factors 3 and 7 deficient (IRF3/7^-/-^) mice.

## Methods

### *Aedes albopictus* mosquitoes

A colony of *Aedes albopictus* was established from eggs collected on Hammond Island (Torres Strait, Australia) in May 2014, with additional wild-caught mosquitoes included in 2015. Generations 94–98 (counted from 2014) were used in the experiments described herein. The colony was maintained in a climate-controlled insectary at the QIMR Berghofer Medical Research Institute at 27 °C, 70 % relative humidity and 12:12 h light:dark cycling with 30 min crepuscular periods. Eggs were hatched by flooding in rainwater. Larvae were reared in rain water in plastic trays at densities of ≈ 500 larvae per tray. Larvae were fed ground TetraMin Tropical Flakes fish food (Tetra, Melle, Germany) ad libitum. Pupae were collected and placed in a container of rainwater inside a 30 × 30 × 30 cm cage (BugDorm, MegaView Science Education Services Co., Taichung, Taiwan). The cage was provided with 10 % sucrose solution on cotton wool pledgets. Prior to feeding, mosquitoes (5–6 day-old) were deprived of sucrose solution for 24 h. Female mosquitoes were sampled from the cage by placing a bottle of hot water beside one of the cage walls and aspirating females that were probing against the bottle. Female mosquitoes (80–110) were added to each 750 ml plastic containers with gauze lids.

### Membrane feeding

Mosquitoes (80–110 per CHIKV dose) were offered defibrinated sheep blood for 1 h (Life Technologies, Mulgrave, VIC, Australia) via a bovine ceacum membrane using an artificial feeding apparatus (kept at 37 °C) as described [7]. The blood meals contained 5-fold serial dilutions of CHIKV stock (LR2006-OPY1; GenBank KT449801 [8] prepared as described [9]) starting at a 1 in 5 dilution. Blood meal titres were determined by CCID_50_ assays on blood meal samples taken before and after mosquito feeding. Engorged mosquitoes (feeding rate range 15–50 %), anaesthetized with CO_2_ and placed on a Petri dish on wet ice, were collected and maintained in an environmental chamber (Panasonic, Osaka, Japan) set at 28 °C, 75 % humidity and 12:12 h day:night light schedule with 30 min dawn:dusk periods.

### Feeding on CHIKV infected mice

Female C57BL/6 J mice (6–8 weeks) were purchased from Animal Resources Center (Canning Vale, WA, Australia) and were inoculated by needle injection with 2 × 10^2^ or 2 × 10^4^ CCID_50_ of C6/36-derived Reunion Island isolate of CHIKV (LR2006-OPY1; GenBank KT449801 [8]) s.c. into hind feet as described previously [8, 9]. On days 2, 7 or 10 post-infection, mice (*n* = 3 per dose and time point) were anesthetized for 30 min with a continuous flow of 3 % isoflurane using a Stinger AAS anesthetic specialist machine (Advanced Anaesthesia Specialists, Gladesville, NSW, Australia) and placed over the gauze of the mosquito containers to allow feeding. Engorged mosquitoes were collected and maintained as above.

### Feeding of CHIKV-infected mosquitoes on naïve mice: viraemias, foot measurements and ELISA

Mosquitoes (*n* = 14–22 per mouse), which had taken a CHIKV-infected blood meal via membrane feeding 7/8 days previously, were allowed to feed on anesthetized naïve female C57BL/6 mice and IRF3/7^-/-^ mice (described previously [10]) (*n* = 3 per group), with the numbers of engorged and probing mosquitoes noted. Viraemias were determined by CCID_50_ assays as described [9]. Height and width of feet were measured by digital callipers and expressed as mean of the percentage increases in height x width for each foot as described [8, 10]. Serum anti-CHIKV IgG2c titres were determined by ELISA on day 21 post-infection as described [11].

### Mosquito viral titre determination

Viral titres in each individual mosquito were determined 7 days after the blood meal, at which time infection levels reach a plateau [12, 13]. Individual mosquitoes (anesthetized and collected as above) were placed in 2 ml screw cap vials with 4–5 zirconium silica beads and 500 μl of medium [RPMI 1640, 2 % FBS/FGS, 0.25 μg/ml Amphotericin B (Gibco; Thermo Scientific, Waltham, MA, USA) and 10 mM HEPES]. Mosquitoes were homogenized by shaking tubes for 1 min 30 s in a chilled block using a MiniBeadbeater-96 sample homogenizer (Biospec Products, Bartlesville, OK, USA) followed by centrifugation (twice at 17,000× *g*, 10 min, 4 °C, with tube rotation), and viral titration using CCID_50_ assays as described [9].

### Mosquito immunohistochemistry and staining quantification

Mosquitoes were processed for immunohistochemistry and paraffin sections stained with a mouse anti-CHIKV capsid monoclonal antibody (5.5G9 [14]) and an Alexa Fluor 488 donkey anti-mouse secondary antibody (green), with DNA stained using DAPI (blue). Stained sections were scanned, and staining quantified using Aperio eSlide Manager and ImageScope Viewer software (Aperio). Full details are available in Additional file 1.

### Statistics

Statistical analyses were performed using IBM SPSS Statistics (version19). The non-parametric Spearman’s rank correlation test was used to determine the relationship between blood meal titers offered via membrane feeding and the resulting CHIKV titres in the mosquitos. The non-parametric Kolmogorov-Smirnov test was used to compare salivary gland CHIKV staining densities as differences in variance were > 4 [8].

## Results

### Infection of mosquitoes via membrane feeding versus infected mice

**Fig. 1:**
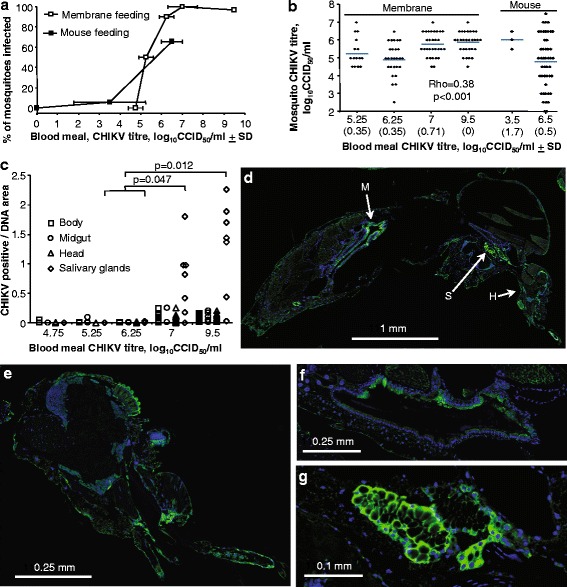
**a** Membrane and mouse feeding of *Aedes albopictus* with different titres of CHIKV. Mosquitoes were fed via artificial membrane with ovine blood containing different titres of CHIKV; the membrane blood meal titres represent the mean (and standard deviation, SD) of before and after feeding titres (i.e. the mean and SD of 2 titre determinations; 30 blood-fed mosquitoes were examined for each CHIKV blood meal titre; limit of detection 2 log_10_CCID_50_/ml). A different batch of mosquitoes were fed on CHIKV infected mice on day 2 post-infection; 2 groups of three mice were inoculated with 2 × 10^2^ or 2 × 10^4^ log_10_CCID_50_/ml CHIKV, with *n* = 49/50 fed mosquitoes for each group. The mouse blood meal titres represent the mean (and SD) viraemia on day 2 (*n* = 3) for each group. **b** CHIKV titres in the mosquitoes. The CHIKV titers of all CHIKV positive mosquitoes from **a** are shown; *blue lines* represent means for each blood meal titre. No CHIKV was detected in any mosquitoes fed with a blood meal titre of 4.75 log_10_CCID_50_/ml. Whole mosquitoes were homogenized in 0.5 ml of medium and titres determined by standard CCID_50_ assays. For membrane fed mosquitoes, a Spearman correlation was performed comparing blood meal titres and mosquito titres, with Rho and p values provided. **c** Quantification of anti-CHIKV staining density. Mosquitoes were fed as in A and 5–8 fed mosquitoes per blood meal dose were examined by immunohistochemistry for CHIKV using an anti-capsid monoclonal antibody and DAPI staining for DNA. Staining areas were quantified by image analysis and expressed as a ratio of CHIKV staining over DAPI staining for each organ/tissue. Mean background staining density in uninfected mosquitoes was 0.004, range 0–0.15). Statistics by Kolmogorov-Smirnov tests: (i) *P* = 0.047, comparing salivary gland staining for mosquitoes given blood meals containing 7 log_10_CCID_50_/ml (*n* = 7) with staining for those given 5.25/6.25 log_10_CCID_50_/ml (staining data for the latter two doses were combined to provide *n* = 4) and (ii) *P* = 0.012, comparing salivary gland staining for mosquitoes given blood meals containing 9.5 log_10_CCID_50_/ml (*n* = 7) with staining for those given 5.25/6.25 log_10_CCID_50_/ml (*n =* 4). **d** Example of whole body section showing IFA staining in: head (H); midgut (M); and salivary glands (S). **e**-**g** High resolution images of IFA staining in head, midgut and salivary glands, respectively

Whether artificial membrane feeding of mosquitoes (usually involving virus inoculated into anti-coagulated bovine or ovine blood [15–17]) accurately recapitulates feeding on viraemic animals (and thus represents a realistic methodology for assessing vector competence) remains a subject for investigation [18–20]. *Aedes albopictus* mosquitoes were fed (i) via membrane feeding using a range of virus titres; and (ii) on mice that had received high and low CHIKV inocula (*n* = 3 per dose) resulting in mean viraemias on day 2 of 6.5 ± 0.5 and 3.5 ± 1.7 log_10_CCID_50_/ml, respectively. The percentage of mosquitoes that became infected increased with the blood meal virus titres, with membrane feeding and feeding on mice providing overlapping and broadly comparable results (Fig. 1a). A threshold effect was evident with titres of > 5 log_10_CCID_50_/ml needed before ≥ 50 % of mosquitoes become infected (Fig. 1a).

### A correlation between blood meal titres and virus levels in mosquitoes

Although a relationship between blood meal titres and the percentage of mosquitoes that become infected is well established [18, 21], the relationship between blood meal virus titres and the resulting virus titres in mosquitoes has, to our knowledge, not been investigated for CHIKV, with a relationship established in some but not other systems [19, 22–24]. The viral titre of each positive mosquito from Fig. 1a was determined, with the results illustrating a significant correlation (Spearman’s correlation, *rho* = 0.38, *P* < 0.001, *n* = 101) between the blood meal titres and the CHIKV titres in the mosquito, although a 4.25 log_10_CCID_50_/ml rise in the former only resulted in a mean ≈ 1 log_10_CCID_50_/ml rise in the latter.

Membrane feeding with 6.25 log_10_CCID_50_/ml and mouse feeding with 6.5 log_10_CCID_50_/ml also produced similar virus titres in the mosquitoes (Fig. 1b), supporting the contention that membrane and mouse feeding provide similar results.

### Immunofluorescent antibody staining of CHIKV in mosquitoes

Using a recently developed monoclonal antibody recognizing the CHIKV capsid protein [14], a group of mosquitoes fed by membrane feeding (as in Fig. 1a, b) were analysed by immunofluorescent antibody staining. The percentage of mosquitoes showing staining above background in at least some area(s) of the different organs/tissues was determined, with broadly similar results for each organ/tissue (Additional file 1: Figure S1). These data correlated well with the data in Fig. 1a and b. However, quantification of the CHIKV staining density (relative to nuclear DNA staining) across the whole organs/tissues, illustrated that pronounced (and significantly increased) staining densities were only observed in mosquitoes fed with blood meals containing viral titres of ≥ 7 log_10_CCID_50_/ml (Fig. 1d). In addition, high staining densities were observed in nearly all salivary glands examined in such mosquitoes (Fig. 1d). High staining densities in salivary glands are perhaps consistent with a recent report of replication of CHIKV in the salivary gland of *Aedes albopictus* [25]. An example of staining of a whole mounted mosquito (Fig. 1e) and the different organs/tissues are shown (Fig. 1f-g).

### No infection of mosquitoes with tissue-associated virus post-viraemia

Infection of mosquitoes by arboviruses in the absence of a detectable viraemia has been reported [26]. After the end of the 4–5 day viraemic period, high titres of replication competent CHIKV persist in mouse foot tissues until day 7 [9], with viral RNA persisting for up to 100 days [8]. Mosquitoes were thus allowed to feed on the feet of mice day 7 post-infection, with the feet of anesthetized mice accessible via the mesh in the lid of the mosquito container. The feet were placed through holes in a piece of paper preventing feeding on the mouse body. Although the mean feet tissue titres on day 7 were 6.1 ± 0.9 log_10_CCID_50_/mg (*n* = 3 mice), none of the 85 fed mosquitoes were infected (data not shown). A repeated experiment day 10 post-infection also resulted in none of the 86 fed mosquitoes becoming infected (data not shown).

### Mosquito to mouse transmission

Transmission of CHIKV from mosquitoes to mice has, to our knowledge, only been reported for wild-type suckling mice [13, 27]. Mosquito-mediated infection of interferon receptor 3 and 7 deficient (IRF3/7^-/-^) mice has been reported for dengue virus [28], with IRF3/7^-/-^ mice also highly susceptible to CHIKV infection due to their inability effectively to generate type I interferon responses [10]. Mosquitoes were membrane fed on blood meals (with a CHIKV titre of 7.5 ± 0.35) and left for 8 days (and allowed to lay eggs) and were then fed on the shaved belly area of wild-type C57BL/6 and IRF3/7^-/-^ mice (*n* = 3 per strain). Whole body CHIKV titres in 10 of these mosquitoes was determined (as in Fig. 1b) to be 6.15 ± 0.58 (SD) log_10_CCID_50_/ml, with all 10 mosquitoes CHIKV positive.

Only a small number of mosquitoes took a detectable second blood meal (Table 1), although 1–4 mosquitoes per mouse were seen to probe, with probing previously reported to result in arbovirus inoculation [29]. All mice became viraemic within 2 days and developed CHIKV-specific IgG responses (confirming infection) (Table 1). IRF3/7^-/-^ also developed swollen feet (Table 1; Additional file 1: Figure S2), with 2/3 mice requiring euthanasia (as described previously [10]).

**Table 1:** Transmission of CHIKV from mosquitoes to mice

Mice	Confirmed mosquitoes fed	Viraemia day 2 (log_10_CCID_50_/ml)	Peak % foot swelling R & L	Mortality; day post-feeding	IgG2c anti-CHIKV response
C57BL/6J					
1	3	7.5	nd	Alive	+^a^
2	2	7.5	nd	Alive	+^a^
3	1	7.5	nd	Alive	+^a^
IRF3/7^-/-^					
1	1	12.5	101 (d 9) 14 (d 13)	13	+^b^
2	1	10	17 (d 13) 46 (d 13)	14	+^b^
3	1	10.5	59 (d 7) 70 (d 7)	Alive	+^b^

*Abbreviation*: *nd* not detected, *R & L* right and left foot

^a^OD >17-fold higher than background for 1/20 dilutions of sera collected day 21 post-feeding

^b^OD >10-fold higher than background for 1/20 dilutions of sera collected upon euthanasia or day 21 post-feeding

In a second experiment, mosquitoes fed with a blood meal of 5.9 ± 0.9 (SD) log_10_CCID_50_/ml, after 7 days were allowed to feed on three naïve C57BL/6 mice. Although more mosquitoes were used in this experiment and 10–17 mosquitoes per mouse took a detectable second blood meal, no infection of C57BL/6 mice was detected (data not shown). This is consistent with the data in Fig. 1d showing that mosquitoes fed on a blood meal containing ≤ 6.25 log_10_CCID_50_/ml of CHIKV failed to show significant levels of CHIKV in salivary glands.

## Discussion

Herein we show for CHIKV and *Aedes albopictus* that provision of blood meals via membrane feeder or via viraemic mice provided overlapping and broadly comparable results, supporting the view that membrane feeding represents a credible method for assessing vector competence [19, 20]. Furthermore, only blood-borne virus appeared able to transmit to mosquitoes, with (post-viraemic) tissue-associated virus unable to transmit, perhaps because it is not efficiently imbibed and/or because neutralising antibodies (present day 7 post-infection [8]) prevent infection of mosquitoes.

Blood meal titres needed to be > 5 log_10_CCID_50_/ml before more than ≥ 50 % of mosquitoes become infected. However, only blood meal titres of ≥ 7 log_10_CCID_50_/ml resulted in significant levels of virus in salivary glands, with direct evidence for CHIKV replication in salivary glands recently provided [25]. Virus in the salivary glands is clearly a key requirement for onward transmission to vertebrate hosts, and our observations are consistent with the notion of a dose-dependent barrier to salivary gland infection [30]. Although comparisons are complicated by different methods for quantifying CHIKV titres, the requirement for high titres blood meal (10^7^ pfu/ml) for infecting a high percentage of *Aedes albopictus* mosquitoes with CHIKV has been reported previously [13, 21], with 10^7.5^ pfu/ml used in another study [12]. Such high titre blood meals were also used to infect mosquitoes that were subsequently used to infect suckling mice [13]. CHIKV viraemias do reach high levels in both mice and humans, albeit only for a few days [10, 31, 32]. However, the full spectrum of inter-relationships between blood meal titres and overt salivary gland infection, and the influence of *inter alia* time post-feeding, temperature and the presence of other infection(s) in the mosquito, remain to be explored.

This paper represents the first report of infection of adult wild-type mice and IRF3/7^-/-^ mice by CHIKV-infected mosquitoes, providing a convenient new model for studying transmission of CHIKV from mosquitoes to mammalian hosts [33–37]. Mosquito-mediated infection of IRF3/7^-/-^ mice with CHIKV also resulted in joint swelling, an arthritic manifestation often seen in symptomatic human CHIKV infections [1]. CHIKV disease manifestations are often more severe in the elderly and the very young [1], populations with compromised type I interferon and/or IRF7 responses [38–42]. The rapid appearance of the CHIKV viraemia (within 2 days), in both wild-type and IRF3/7^-/-^ mice post-mosquito feeding, recapitulates the often short incubation period seen for CHIKV infections in humans [1]. The results also argue that the main barrier to transmission is the presence of significant levels of virus in the mosquito salivary glands, rather than the existence of a resistance barrier in adult mice [43, 44].

## Conclusion

Feeding *Aedes albopictus* mosquitoes CHIKV infected blood meals, via a membrane feeder or via infected mice, did not result in marked differences in mosquito infection rates, supporting the view that membrane feeding is a credible method for assessing vector competence. For mosquito salivary glands to become clearly infected, the blood meal titres needed to be ≈ 1–2 logs higher than the titres required simply to infect the mosquitoes. Mosquitoes fed the high titre blood meals were able efficiently to transmit CHIKV to adult mice. The results argue against the presence of a resistance barrier in adult mice and provide a laboratory model for studying transmission of CHIKV from mosquitoes to mammals.

## Additional file

Additional file 1: Figure S1. The percentage of mosquitoes where some positive staining for the indicated organs/tissues was evident is shown; from the experiment described in Fig. 1c-g. Quantification of staining density for this experiment is shown in Fig. 1c. **Figure S2.** Image of foot swelling in IRF3/7^-/-^ mice. Detailed methods: mosquito immunohistochemistry and quantification. (PDF 71 kb)

## Abbreviations

CCID: Cell culture infective dose
CHIKV: Chikungunya virus
IRF: Interferon response factor
