# Supplementary material for: Chikungunya virus transmission between Aedes albopictus and laboratory mice
Source: Parasit Vectors. 2016 Oct 19;9:555. doi: 10.1186/s13071-016-1838-1 (PMC5069946; doi:10.1186/s13071-016-1838-1)
Supplement: Additional file 1: Figure S1. — The percentage of mosquitoes where some positive staining for the indicated organs/tissues was evident is shown; from the experiment described in Fig. 1c-g. Quantification of staining density for this experiment is shown in Fig. 1c. Figure S2. Image of foot swelling in IRF3/7-/- mice. Detailed methods: mosquito immunohistochemistry and quantification. (PDF 71 kb) [file 13071_2016_1838_MOESM1_ESM.pdf]

Additional file 1

Fig. S1

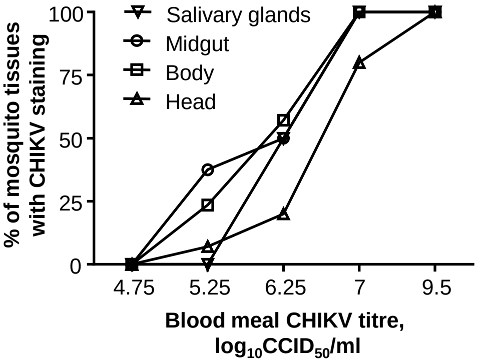

Fig. S2

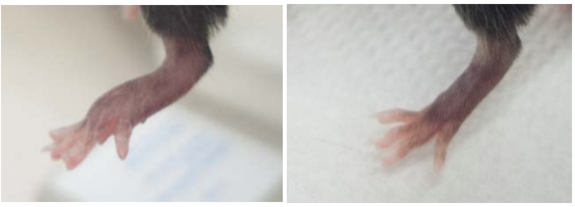

Infected IRF3/7$^{-/-}$ Uninfected IRF3/7$^{-/-}$

Detailed methods; mosquito immunohistochemistry and quantification. Mosquitoes were fixed in 4% paraformaldeyde, 0.5% Triton X and embedded in paraffin using standard procedures. Paraffin sections (3-4 µM) were fixed to Menzel Superfrost Plus adhesive slides and air-dried overnight at 37°C. Slides were dewaxed and rehydrated through descending graded alcohol series and antigen retrieval performed in Dako Target Retrieval Solution pH 9.0 (Dako corporation, Carpinteria, CA, USA) for 15 min at 100°C in a Biocare Medical Decloaking chamber (Biocare Medical, Walnut Creek, CA, USA). Slides were washed in three changes of PBS with 0.02% Tween 20 and blocking of non-specific antibody binding was performed by incubating slides in Biocare Medical Background Sniper with 2.0% BSA for 2 hr. This solution was removed from the slide and replaced with a solution comprising 3 parts primary antibody (5.5G9 tissue culture supernatant) and 1 part Da Vinci Green diluent (Biocare Medical) and incubated overnight at room temperature. Slides were washed in three changes of PBS with 0.02% Tween 20, followed by Alexa Fluor 488 donkey anti-mouse secondary antibody (Molecular Probes) and DNA staining with DAPI (blue) and mounted in Prolong Gold fluorescence mounting media (Molecular Probes; Eugene, OR, USA). Slides were scanned using an Aperio ScanScope FI slide scanner (Aperio Technologies, Vista, CA, USA) at an apparent magnification of 20. DAPI excitation was 345 nm and emission collection was 455 nm with 0.1 s exposure. AlexaFluor® 488 excitation was 495 nm and emission was 519 nm with a 0.32 s exposure. Staining areas were quantified using Aperio eSlide Manager and ImageScope Viewer software (Aperio). Analysis regions were created by circumscribing mosquito organs/tissues (midgut, salivary glands, head and body). Analysis was performed by dividing the area of Alexa-488 positive pixels by the area of DAPI positive pixels to provide an approximate estimate of the proportion of cells positive for CHIKV. The analysis regions for bodies were circumscribed by the margins of the abdomen and thorax and excluded any regions covered by midgut or salivary gland tissue.
